# Transarterial Chemoembolization With or Without Systemic Therapy for Unresectable Hepatocellular Carcinoma: A Retrospective Comparative Study

**DOI:** 10.1002/cam4.70633

**Published:** 2025-02-05

**Authors:** Chengxiang Guo, Weiran Du, Yiwen Chen, Wenbo Xiao, Ke Sun, Yan Shen, Min Zhang, Jian Wu, Shunliang Gao, Jun Yu, Risheng Que, Xing Xue, Xueli Bai, Tingbo Liang

**Affiliations:** ^1^ Department of Hepatobiliary and Pancreatic Surgery The First Affiliated Hospital, Zhejiang University School of Medicine Hangzhou China; ^2^ Zhejiang Provincial Key Laboratory of Pancreatic Disease Hangzhou China; ^3^ Zhejiang Provincial Clinical Research Center for the Study of Hepatobiliary & Pancreatic Diseases Hangzhou China; ^4^ Cancer Center Zhejiang University Hangzhou China; ^5^ Department of Radiology The First Affiliated Hospital, Zhejiang University School of Medicine Hangzhou China; ^6^ Department of Pathology The First Affiliated Hospital, Zhejiang University School of Medicine Hangzhou China

**Keywords:** combination therapy, hepatocellular carcinoma, survival, transarterial chemoembolization

## Abstract

**Introduction:**

Standard treatments provide limited benefits for patients with intermediate‐ or advanced‐stage hepatocellular carcinoma (HCC). This retrospective observational study aimed to assess the potential improvements in outcomes associated with systemic therapies in patients receiving transarterial chemoembolization (TACE) for initially unresectable HCC.

**Methods:**

Between February 2019 and March 2023, we reviewed patients diagnosed with intermediate‐to‐advanced HCC who were treated with either TACE or TACE combined with antiangiogenic agents and immune checkpoint inhibitors (combination therapy) as their initial treatment. To address potential confounding biases, patients were further stratified into surgical and non‐surgical cohorts, and separate analyses were conducted. The primary endpoints were progression‐free survival (PFS) and overall survival (OS), with safety profiles also evaluated.

**Results:**

Among 279 patients with initially unresectable intermediate or advanced HCC, 156 successfully underwent curative‐intent liver resection after preoperative treatments (TACE group, *n* = 69; combination group, *n* = 87), while 123 patients continued with non‐surgical treatments (TACE group, *n* = 31; combination group, *n* = 92). After propensity score matching, 26 matched patient pairs were generated within the non‐surgical cohort. The combination group exhibited significantly improved PFS in non‐surgical patients compared with the TACE group (9.4 vs. 7.2 months, *p* = 0.043). Cox proportional hazards analysis further confirmed that combination therapy was associated with improved PFS (hazard ratio = 0.476, 95% confidence interval: 0.257–0.883, *p* = 0.019). For surgical patients exceeding the up‐to‐seven criteria, the combination group demonstrated superior median PFS (18.0 vs. 14.6 months, *p* = 0.03) and OS (not reached vs. 50.1 months, *p* = 0.049) compared with the TACE group. Adverse events were manageable, with no treatment‐related fatalities reported.

**Conclusion:**

Combination therapy with TACE demonstrated enhanced survival benefits for patients with intermediate to advanced HCC, particularly in surgical patients with higher tumor burdens.

## Graphical abstract

Transarterial chemoembolization in combination with systemic antitumor therapy demonstrated improved survival benefits in patients with intermediate to advanced hepatocellular carcinoma, particularly among surgical patients with higher tumor burden.

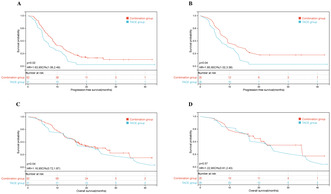

## 1 Introduction

Hepatocellular carcinoma (HCC) is one of the most prevalent and lethal malignancies worldwide, representing a relative 5‐year survival rate of approximately 18% [1, 2]. The Barcelona Clinic Liver Cancer (BCLC) algorithm, which categorizes patients with HCC into five clinical stages, has been extensively employed for treatment allocation and prognosis prediction [3, 4]. Although curative surgical therapies can provide significant survival benefits for early‐stage cases (BCLC stage 0 or A), the majority of patients with HCC are diagnosed at an initially unresectable stage (BCLC Stage B or C). In such cases, transarterial chemoembolization (TACE) or systemic therapy plays a critical role in the management of intermediate or advanced HCC according to current clinical practice [5]. Nevertheless, the efficacy of standard treatment alone remains unsatisfactory.

Recently, recommendations on multimodal treatment regimens for patients with locally advanced HCC have garnered much attention [6, 7]. Numerous clinical studies have explored combining TACE with molecularly targeted or immune therapies, demonstrating promising results with tolerable toxicity for patients with advanced or unresectable HCC [8, 9, 10, 11, 12, 13]. Encouraging improvements in survival have been reported in patients with technically and oncologically unresectable HCC following conversion therapy [14]. Furthermore, several studies have investigated the perioperative effects and oncological outcomes of preoperative TACE combined with antiangiogenic therapy and/or immune checkpoint inhibitors (ICIs) [15, 16, 17]. However, it remains necessary to further explore whether more proactive combination strategies could reduce postoperative recurrence or achieve long‐term tumor control. In addition, it is crucial to identify the most effective preoperative treatments that provide survival benefits for patients with intermediate or advanced HCC.

Therefore, this retrospective research was designed to evaluate the clinical outcomes of TACE alone or in combination with immune and targeted therapy as primary treatment for patients with intermediate to advanced stage HCC.

## 2 Methods

### 2.1 Study Design and Patients

This study was reviewed and approved by the research ethics committee of our hospital and was conducted in accordance with the principles of the Declaration of Helsinki and the STROCSS criteria [18]. We retrospectively analyzed medical records from 598 consecutive patients with treatment‐naive intermediate or advanced HCC who received either TACE alone or the combination therapy (TACE plus antiangiogenic agents with or without ICIs) at our institution between February 2019 and March 2023. These patients were deemed ineligible for primary surgical resection by our multidisciplinary team (MDT) due to factors such as insufficient residual liver volume, uncontrolled comorbidities, or technical challenges. All treatment decisions were reviewed by the attending physicians and approved by individual patients. This study is registered on ClinicalTrials.gov under the identifier NCT06261138.

Enrolled patients were also met the following criteria: (1) age between 18 and 75 years, (2) histologically or clinically confirmed HCC based on the American Association for the Study of Liver Diseases criteria [19] and Chinese Guidelines for Diagnosis and Treatment of Primary Liver Cancer [20], with no prior antitumor therapy, (3) at least one reproducibly measurable target lesion according to modified Response Evaluation Criteria in Solid Tumors (mRECIST) [21], (4) BCLC stage B or C, and Child‐Pugh liver function of A (score 5–6) to B (score 7), (5) anti‐angiogenic therapy administrated for more than 1 month, with no restrictions on the specific types of systemic agents used, (6) adequate organ and hematologic function.

Exclusion criteria included the following: (1) tumor recurrence after curative resection or ablation or diffuse unresectable HCC, (2) spontaneous rupture or bleeding of HCC, (3) pathologically confirmed cholangiocarcinoma or mixed HCC in cases involving surgical resection, (4) evidence of autoimmune disease, other concomitant malignancies, or prior organ transplantation, (5) an interval of more than 1 month between the initial administration of systemic therapy and the first TACE procedure in the combination therapy group, (6) inability to complete a full treatment cycle due to severe adverse reactions, (7) incomplete imaging or follow‐up information.

### 2.2 TACE Procedure

Patients included in the study underwent either conventional TACE (cTACE) or drug‐eluting beads TACE (DEB‐TACE) procedures, which were performed by experienced interventional radiologists. These decisions were guided by tumor burden, patient tolerance, and individual preferences. The procedures began with the cannulation of the femoral arteries using an arterial catheter sheath via the Seldinger technique under local anesthesia. A 5‐French RH catheter was then advanced into the celiac artery, superior mesenteric artery, and common hepatic artery for angiography to assess tumor location, quantity, size, and vascularity. After confirmation using C‐arm cone‐beam computed tomography (CBCT), chemoembolization was performed through superselective catheterization of the branches of the tumor‐feeding arteries using a 2.8‐French coaxial microcatheter. For cTACE, oxaliplatin, raltitrexed, and an emulsion containing idarubicin and ethiodized oil were infused over a period of 20 min, followed by embolization using blank embolic microspheres. For DEB‐TACE, DC/LC Beads (Biocompatibles, Farnham, Surrey, UK), which serve as both drug carriers and embolic materials, were loaded with doxorubicin hydrochloride and mixed with nonionic contrast medium. Embolization was monitored in real time with the assistance of CBCT, allowing for adjustments during the procedure. The specific dosage of chemotherapy drugs and embolization agents was tailored to achieve effective embolization based on the patient's tumor characteristics, body surface area, and the patient's specific condition. If necessary, additional embolization was performed until slow blood flow or near stasis was observed in the arteries directly feeding the tumor. After the procedure, the catheter sheaths were removed, pressure bands were applied, and standard supportive care was provided [22].

The need for repeated TACE procedures was determined based on objective tumor responses as evaluated by MDT.

### 2.3 Systemic Treatment

Systemic treatment was initiated within 1 month after the initial TACE procedure, depending on the adequate liver function. The anti‐angiogenic agents used in this study primarily included multikinase tyrosine kinase inhibitors, such as sorafenib (Bayer HealthCare, Berlin, Germany), lenvatinib (Eisai, Tokyo, Japan), apatinib (Hengrui Pharmaceuticals, Lianyungang, China), and donafenib (Zelgen Biopharmaceuticals, Suzhou, China), while a small proportion of patients received bevacizumab (Innovent Biologics, Suzhou, China), a vascular endothelial growth factor inhibitor. ICIs consisted of therapies targeting programmed cell death protein 1 and its ligands, including sintilimab (Innovent Biologics, Suzhou, China), toripalimab (Junshi Biosciences, Suzhou, China), camrelizumab (Hengrui Pharmaceuticals, Lianyungang, China), tislelizumab (BeiGene, Shanghai, China), pembrolizumab (Merck Sharp & Dohme, NewJersey, USA), nivolumab (Bristol Myers Squibb, New York, USA), atezolizumab (Roche, Basel, Switzerland), and envafolimab (Alphamab Biopharmaceuticals, Suzhou, China).

The decision to combine systemic treatments was informed by guideline recommendations, reported efficacy and safety data, and MDT discussions. The selection of specific medications was made collaboratively with the patient, considering the pros and cons of each option, drug availability, and medical insurance or charity policies. Initial dosages and frequencies of systemic medications were administered according to the drug manufacturer's instructions. Dose reduction or treatment interruption was determined by the physician's judgment and was primarily influenced by treatment‐related side effects or the patient's clinical condition. Medications were discontinued in cases of intolerable toxicity, disease progression, or patient preference.

### 2.4 Surgical Resection

Patients were considered for subsequent curative resection based on MDT evaluations if they met the following criteria: (1) evidence of significant tumor shrinkage or necrosis with a decline in serum tumor markers, (2) technically resectable with clear margins, (3) sufficient liver reserve function and remnant liver volume, and (4) overall good health and willingness to undergo surgery [14]. Hepatectomy was typically performed within 8 weeks following the last TACE treatment, with a 2‐week interruption in systemic therapy. Details regarding the surgical procedure and postoperative management have been described in our previous studies [23]. Additional radiotherapy or intraoperative radiofrequency ablation was applied when clinically feasible. For patients with macrovascular invasion, thrombectomy was performed as required, depending on the location and extent of the tumor thrombus [24, 25, 26].

The pathological analysis included tumor size, number, margins, presence of microvascular invasion (mVI), and the percentage of residual viable tumor, all of which were reviewed by experienced pathologists [27]. Postoperative adjuvant therapy was customized based on tumor pathology, preoperative treatment response, and patient condition.

### 2.5 Assessments and Follow‐Up

At the initial diagnosis and within 4–8 weeks after each TACE treatment session, comprehensive laboratory tests and imaging examinations, including enhanced computed tomography or nuclear magnetic resonance imaging, were routinely conducted. During imaging assessments, portal vein invasion was categorized as Vp3 (invasion of the first‐order branches) or Vp4 (invasion of the main trunk or contralateral branches), in accordance with widely accepted staging conventions. Similarly, hepatic vein invasion was defined as Vv2 (invasion of the major hepatic vein branch) or Vv3 (invasion of the inferior vena cava). Treatment response, classified as complete response (CR), partial response, stable disease, or progressive disease (PD), was assessed and reviewed by experienced diagnostic radiologists in accordance with the mRECIST [21]. The best preoperative tumor response was recorded as the final evaluation result for patients who underwent surgical resection.

The established treatment plan required modification if patients experienced PD or intolerable adverse effects, as determined through MDT discussions. Alternatively, it was recommended to either continue the current therapy or consider radical resection depending on the specific condition. For patients with progression or resection, regular follow‐up assessments were carried out every 2–3 months thereafter to ascertain the types of recurrence (based on angiographic and/or radiologic findings) and survival status. The choice of subsequent treatments was also guided by MDT recommendations and the patients' preferences.

Treatment‐related adverse events (TRAEs) or postoperative complications were documented in the patients' medical records or ascertained through follow‐up inquiries, with the severity graded using the National Cancer Institute Common Terminology Criteria for Adverse Events (version 5.0) or the Clavien‐Dindo classification [28]. For TRAEs graded 1 to 2, which were promptly managed with favorable recovery, treatment regimens remained unaltered. In the event of TRAEs graded ≥ 3 or persistent adverse events, dose reduction or treatment suspension was required until symptoms alleviated to grade 1 or 2.

Progression‐free survival (PFS) was the primary endpoint, defined as the time from the initiation of TACE treatment to the first documented disease progression (in non‐surgical patients), tumor recurrence (in surgical patients), or death, whichever occurred first. Overall survival (OS) was the secondary endpoint of our study. For all patients, OS was defined as the duration from the initiation of the first treatment (TACE or TACE combined with systemic therapy) until all‐cause death. Patients who remained alive without observed progression or recurrence were censored at their last follow‐up date, which concluded in October 2023.

### 2.6 Statistical Analysis

Patient baseline information, treatment course, perioperative parameters, and disease progress were collected and analyzed retrospectively. Categorical and continuous variables were compared using the Chi‐squared test and Student's t‐test, respectively. Kaplan–Meier curves were used to illustrate the survival outcomes among different groups, and the significance of differences was assessed through the log‐rank test. All statistical analyses were conducted using SPSS statistical software (Version 23.0, IBM Corp., Armonk, NY, USA), and a two‐sided *p‐*value < 0.05 was considered statistically significant.

Recognizing the potential bias associated with surgical interventions, we stratified our study population into “non‐surgical” and “surgical” groups. To mitigate the selection bias and minimize the impact of confounding factors within the surgical and non‐surgical patients, two distinct statistical approaches were used to comprehensively explore the association between treatment modalities (TACE monotherapy or combination therapy) and survival outcomes. For the non‐surgical group, propensity score matching (PSM) analysis was conducted by incorporating the following covariates into the logistic regression model: age, AFP level, etiology, Child‐Pugh class, tumor number and size, tumor distribution, presence or absence of macrovascular invasion and extrahepatic metastasis, BCLC stage, and concurrent treatment. The caliper width was set at 0.02, ensuring a one‐to‐one match through the nearest‐neighbor method without replacement between the two groups.

For the surgical group, a stratified survival analysis based on the up‐to‐seven criteria [29] was used to balance differences in tumor burden and control for the potential influence of surgical interventions on the study outcomes. Subsequently, univariate and multivariate Cox proportional hazards regression models were applied to identify prognostic factors for survival outcomes, using the coefficients and significance levels of each variable.

## 3 Results

**FIGURE 1:**
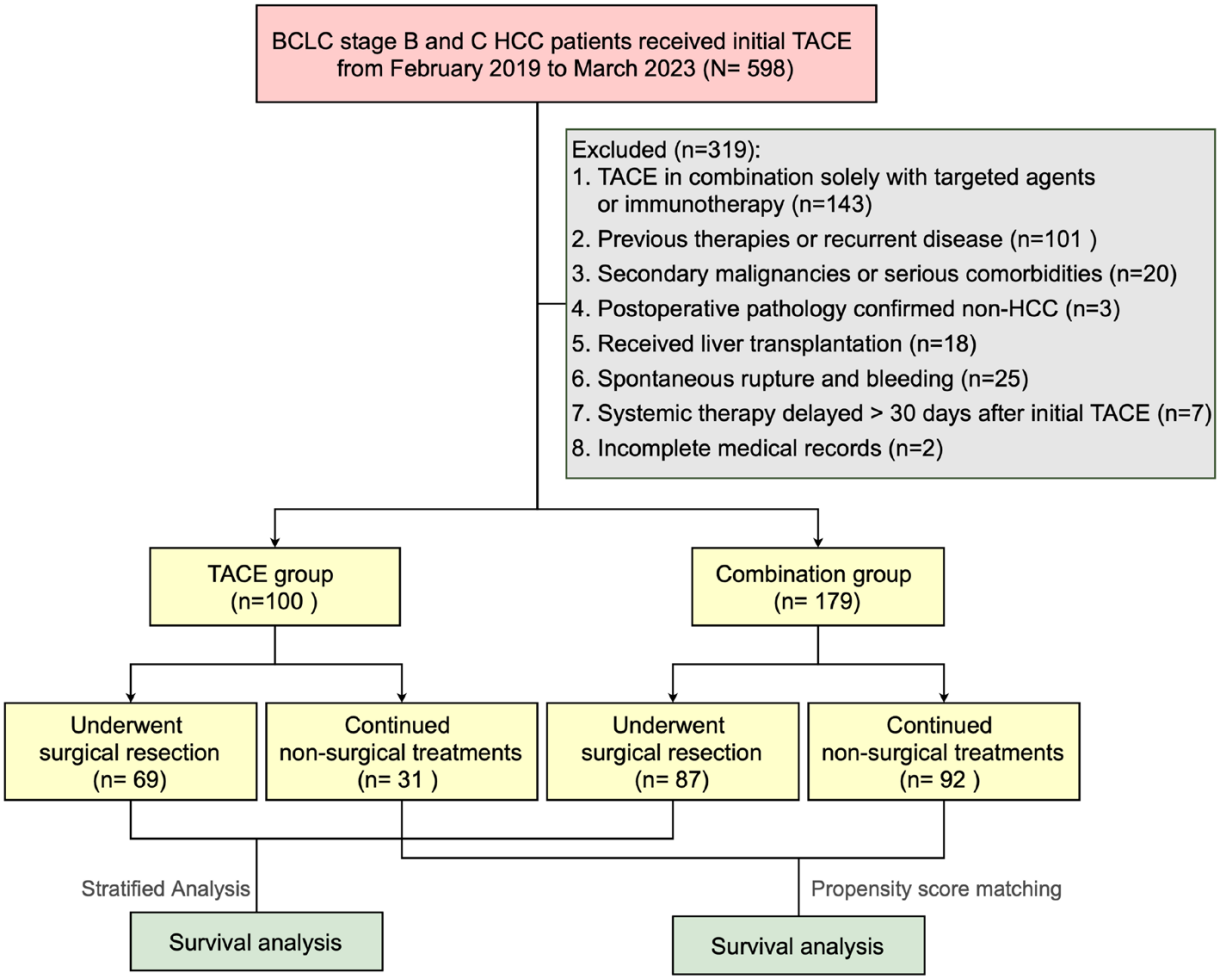
Flowchart of study participant inclusion for analysis. BCLC, Barcelona Clinic Liver Cancer; HCC, hepatocellular carcinoma; TACE, transarterial chemoembolization.

From February 2019 to March 2023, 598 consecutive patients with treatment‐naive HCC who underwent TACE were retrospectively screened. After applying exclusion criteria, a total of 279 patients with initially unresectable intermediate‐advanced HCC were enrolled in the analysis, including 100 patients who received TACE monotherapy (TACE group) and 179 patients who received TACE combined with anti‐angiogenic therapy and ICIs (combination group). Of these, 156 patients successfully underwent subsequent curative‐intent liver resection after preoperative treatments, comprising 69 from the TACE group and 87 from the combination therapy group (*p* < 0.01). Conversely, 31 patients in the TACE group and 92 patients in the combination group continued non‐surgical treatments due to tumor‐related factors or personal choice (shown in Figure 1). The cohort was further divided into non‐surgical and surgical segments for separate analyses.

### 3.1 Characteristics and Treatments of Non‐Surgical Patients

In the TACE group, the median age was 63 years (range, 34–75), with 16.1% showing hepatic vein tumor thrombosis—highlighting a significant difference compared to the combination group. In the combination group, maximum tumor diameter (8.3 cm vs. 8.1 cm, *p* = 0.198), portal vein tumor thrombosis rate (66.3% vs. 54.8%, *p* = 0.252), and extrahepatic metastasis rate (28.3% vs. 16.1%, *p* = 0.178) were slightly higher than those in the TACE group, but without statistical significance. The two groups had comparable proportions of patients undergoing repeated TACE treatments, with 67.7% in the TACE group and 66.3% in the combination group. After PSM, 26 non‐surgical patients from the combination group were matched to 26 non‐surgical patients from the TACE group, resulting in balanced baseline characteristics and staging between the two groups (All *p* > 0.05, Table 1).

**TABLE 1:** Clinical characteristics of non‐surgical patients.

Characteristics	General cohort			PSM cohort		
	TACE (*n* = 31)	Combination (*n* = 92)	*p*	TACE (*n* = 26)	Combination (*n* = 26)	*p*
Age, years, median (IQR)	63 (51–68)	56 (50–62)	0.016	63.5 (52–68.3)	58 (50.8–64.0)	0.241
Gender *n* (%)						
Male	26 (83.9)	84 (91.3)	0.244	22 (84.6)	21 (80.8)	0.714
Female	5 (16.1)	8 (8.7)		4 (15.4)	5 (19.2)	
Chronic HBV infection						
Positive	26 (83.9)	76 (82.6)	0.872	21 (80.8)	23 (88.5)	0.442
Negative	5 (16.1)	16 (17.4)		5 (19.2)	3 (11.5)	
Baseline AFP, ng/mL						
≥ 400	11 (35.5)	47 (51.1)	0.132	9 (34.6)	11 (42.3)	0.569
< 400	20 (64.5)	45 (48.9)		17 (65.4)	15 (57.7)	
Child Pugh						
A	26 (83.9)	79 (85.9)	0.785	24 (92.3)	22 (84.6)	0.385
B	5 (16.1)	13 (14.1)		2 (7.7)	4 (15.4)	
Tumor number^a^						
≤ 2	15 (48.4)	41 (44.6)	0.712	12 (42.6)	13 (50)	0.781
> 2	16 (51.6)	51 (55.4)		14 (53.8)	13 (50)	
Largest tumor size^a^, cm, median (IQR)	8.1 (4.3–9.7)	8.3 (5.5–11.8)	0.198	8 (4–9.8)	7.7 (4.5–9)	0.985
Portal vein tumor thrombosis						
Vp2‐4	17 (54.8)	61 (66.3)	0.252	13 (50)	16 (61.5)	0.402
Absent	14 (45.2)	31 (33.7)		13 (50)	10 (38.5)	
Hepatic vein tumor thrombosis						
Vv2‐3	5 (16.1)	37 (40.2)	0.014	4 (15.4)	9 (34.6)	0.109
Absent	26 (83.9)	55 (59.8)		22 (84.6)	17 (65.4)	
Extrahepatic metastasis						
Present	5 (16.1)	26 (28.3)	0.178	4 (15.4)	5 (19.2)	0.714
Absent	26 (83.9)	66 (71.7)		22 (84.6)	21 (80.8)	
BCLC stage						
B	10 (32.3)	22 (23.9)	0.360	9 (34.6)	8 (30.8)	0.768
C	21 (67.7)	70 (76.1)		17 (65.4)	18 (69.2)	
TACE sessions						
1	10 (32.3)	31 (33.7)	0.883	8 (30.8)	13 (50.0)	0.158
≥ 2	21 (67.7)	61 (66.3)		18 (69.2)	13 (50.0)	
Concomitant radiotherapy						
Yes	6 (19.4)	22 (23.9)	0.601	6 (23.1)	7 (26.9)	0.749
No	25 (80.6)	70 (76.1)		20 (76.9)	19 (73.1)	

Abbreviations: AFP, alpha‐fetoprotein; BCLC, barcelona clinic liver cancer; HBV, hepatitis B virus; IQR, interquartile range; PSM, propensity score matching; TACE, transarterial chemoembolization; Vp2‐4, tumor thrombus involving the portal vein: Vp2 (second‐order branch), Vp3 (first‐order branch), Vp4 (main trunk/contralateral branch); Vv2‐3, tumor thrombus involving the major hepatic vein (Vv2) or inferior vena cava (Vv3).

^a^ Number or size of initially diagnosed tumors.

The TACE group underwent a total of 73 cycles of TACE procedures, while the combination group received 195 cycles. There were no significant differences in the total number or types of TACE treatments between the two groups. However, a statistical difference was observed in the duration of treatments before disease progression (6.1 months vs. 8.4 months, *p* = 0.05). In the combination group, Lenvatinib (72, 78.3%) remained the primary choice among targeted therapies, whereas Tislelizumab (27, 29.3%), Sintilimab (23, 25%), and Envolizumab (19, 20.7%) were commonly used in immunotherapy. After PSM, treatment‐related information became comparable between the two groups (Table S1).

### 3.2 Survival Analysis for Non‐Surgical Patients

The median follow‐up duration for the non‐surgical patients was 16.4 (range, 2.4–43.6) months. At the last follow‐up date, a total of 30 (96.8%) patients in the TACE group and 77 (82.6%) in the combination group experienced disease progression, metastasis, or mortality.

**FIGURE 2:**
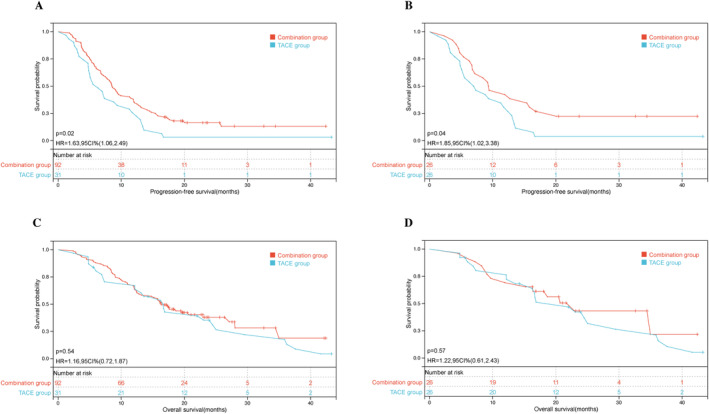
Kaplan–Meier analyses of survival outcomes of non‐surgical patients between the TACE group and combination group before and after propensity score matching (PSM). (A) Progression‐free survival before PSM, (B) progression‐free survival after PSM, (C) overall survival before PSM, and (D) overall survival after PSM. TACE, transarterial chemoembolization.

In both the unmatched cohort (8.6 months [95% confidence interval {CI}, 7.37–9.69] vs. 6.3 months, [95% CI, 4.25–8.35], hazard ratio [HR] = 0.74, *p* = 0.02) and the PSM‐matched cohort (9.4 months [95% CI, 5.0–13.67] vs. 7.2 months, [95% CI, 2.89–11.06], HR = 0.76, *p* = 0.043), patients in the combination group exhibited significantly longer PFS compared with those in the TACE group [Figure 2A,B]. The 1‐year PFS rates before and after PSM were 37.0% and 42.3% in the combination therapy group and 25.8% % and 30.8% in the TACE group, respectively. The median OS of the TACE group was 16.6 months (95% CI: 12.50–20.76), comparable to the combination group, which showed 16.4 months (95% CI: 13.42–19.32), with an HR of 1.02 (*p* = 0.517). For PSM patients, the median OS was also similar between the combination group (22.9 months [95% CI, 15.63–30.23]) and the TACE‐alone group (22.0 months [95% CI, 12.37–31.57]), with an HR of 0.82 (*p* = 0.552), showing no statistical significance [Figure 2C,D].

We also examined the disease progression and post‐progression treatments for all non‐surgical patients. There was no significant difference in the type of progression (intrahepatic disease progression or extrahepatic metastasis) between the two groups. Among those who experienced disease progression, 17 patients (56.7%) in the TACE group and 26 patients (33.8%) in the combination group continued to receive TACE combined with either first‐ or second‐line systemic drugs. In addition, seven patients (9.1%) in the combination group opted to enroll in clinical trials. Details of the treatment regimens provided to patients after tumor progression are presented in Table S1.

Univariate and multivariate Cox regression analyses conducted in the entire non‐surgical patient cohort revealed that TACE treatment (HR = 1.915, 95% CI: 1.224–2.996, *p* = 0.004) and the largest tumor size (HR = 1.069, 95% CI: 1.019–1.122, *p* = 0.006) were independent risk factors associated with reduced PFS. Subsequent multivariate analysis within the PSM cohort yielded similar results, identifying TACE monotherapy (HR = 2.101, 95% CI: 1.133–3.897, *p* = 0.019) and a larger baseline tumor diameter (HR = 1.120, 95% CI: 1.023–1.226, *p* = 0.014) as prognostic factors linked to inferior PFS (Table 2).

**TABLE 2:** Univariate and multivariate Cox regression analysis of PFS in the non‐surgical cohort.

Variables	General cohort			PSM cohort		
	Univariate analysis (*p*)	Multivariate analysis		Univariate analysis (*p*)	Multivariate analysis	
		Hazard ratio (95% CI)	*p*		Hazard ratio (95% CI)	*p*
Treatment, TACE/combination	**0.026**	0.522 (0.334–0.817)	**0.004**	**0.046**	0.476 (0.257–0.883)	**0.019**
Age, years, ≤ 65/> 65	0.910			0.852		
Gender, male/female	0.322			0.375		
Chronic HBV infection, positive/negative	0.338			0.308		
AFP, ng/mL, < 400/≥ 400	0.337			0.643		
Child–Pugh grade, A/B	0.459			0.438		
Tumor number^a^, ≤ 2/> 2	0.829			0.311		
Tumor distribution, uni‐lobar/ bi‐lobar	0.248			0.435		
Largest tumor size^a^	**0.034**	1.069 (1.019–1.122)	**0.006**	**0.037**	1.120 (1.023–1.226)	**0.014**
Portal vein tumor thrombosis, Vp2‐4/absent	0.467			0.911		
Hepatic vein tumor thrombosis, Vv2‐3/absent	0.110			0.713		
Extrahepatic metastasis, present/absent	0.118			0.634		
BCLC stage, B/C	0.785			0.900		
TACE sessions, 1/≥ 2	0.725			0.907		
Concomitant radiotherapy, yes/no	0.222			0.313		

Abbreviations: AFP, alpha‐fetoprotein; BCLC, barcelona clinic liver cancer; CI, confidence interval; HBV, hepatitis B virus; PFS, progression‐free survival; PSM, propensity score matching; TACE, transarterial chemoembolization; Vp2‐4, tumor thrombus involving the portal vein: Vp2 (second‐order branch), Vp3 (first‐order branch), Vp4 (main trunk/contralateral branch); Vv2‐3, tumor thrombus involving the major hepatic vein (Vv2) or inferior vena cava (Vv3).

Bold values indicate statistically significant *p* *<* 0.05.

^a^ Number or size of initially diagnosed tumors.

### 3.3 Characteristics and Treatments of Surgical Patients

All surgical patients were classified as Child‐Pugh A, while the combination group included a higher proportion of patients with advanced HCC (62.1% vs. 40.6%, *p* = 0.008) and exhibited greater tumor burdens (Table 3). The median size of the largest tumor nodule was 6.0 cm in the TACE group compared with 8.9 cm in the combination group (*p* = 0.007). In addition, patients in the combination group presented with elevated baseline AFP levels and a higher incidence of hepatic vein tumor thrombosis. No significant differences were observed in baseline tumor numbers or portal vein tumor thrombosis between the two groups. A similar proportion of patients in the TACE and combination groups underwent concomitant radiotherapy (17.2% vs. 10.1%, *p* = 0.206). However, a significantly greater percentage of patients in the combination group received multiple preoperative TACE sessions (50.6% vs. 17.4%, *p* < 0.001) and underwent postoperative adjuvant treatments (93.1% vs. 68.1%, *p* < 0.001).

**TABLE 3:** Clinical characteristics of surgical patients.

Characteristics	TACE (*n* = 69)	Combination (*n* = 87)	*p*
Age, years, median (IQR)	61 (54–67)	52 (45–59)	< 0.001
Gender, *n* (%)			
Male	61 (88.4)	78 (89.7)	0.804
Female	8 (11.6)	9 (10.3)	
Chronic HBV infection			
Positive	55 (79.7)	71 (81.6)	0.765
Negative	14 (20.3)	16 (18.4)	
Baseline AFP, ng/mL			0.042
< 400	43 (62.3)	40 (46.0)	
≥ 400	26 (37.7)	47 (54.0)	
Tumor number^a^, median (IQR)	2 (2–3)	2 (1–3)	0.289
Largest tumor size^a^, cm, median (IQR)	6 (4–9.2)	8.9 (5–11.8)	0.007
Portal vein tumor thrombosis			0.121
Vp2‐4	21 (30.4)	37 (42.5)	
Absent	48 (69.6)	50 (57.5)	
Hepatic vein tumor thrombosis			0.006
Vv2‐3	15 (21.7)	37 (42.5)	
Absent	54 (78.3)	50 (57.5)	
BCLC stage			
B	41 (59.4)	33 (37.9)	0.008
C	28 (40.6)	54 (62.1)	
Preoperative TACE sessions			< 0.001
1	57 (82.6)	43 (49.4)	
≥ 2	12 (17.4)	44 (50.6)	
Concomitant radiotherapy			0.206
Yes	7 (10.1)	15 (17.2)	
No	62 (89.9)	72 (82.8)	
Adjuvant therapy			< 0.001
Present	47 (68.1)	81 (93.1)	
Absent	22 (31.9)	6 (6.9)	

Abbreviations: AFP, alpha‐fetoprotein; BCLC, barcelona clinic liver cancer; HBV, hepatitis B virus; IQR, interquartile range; TACE, transarterial chemoembolization; Vp2‐4, tumor thrombus involving the portal vein: Vp2 (second‐order branch), Vp3 (first‐order branch), Vp4 (main trunk/contralateral branch); Vv2‐3, tumor thrombus involving the major hepatic vein (Vv2) or inferior vena cava (Vv3).

^a^ Number or size of preoperatively diagnosed tumors.

Regarding treatment details, the median numbers for TACE treatments were 1 (range, 1–4) and 2 (range, 1–4) in the TACE and combination groups, respectively (Table S2). Lenvatinib (69, 79.3%) was the most frequently used anti‐angiogenic agent, while Sintilimab (31, 35.6%) was the primary choice among immunotherapies. The median duration between the initial TACE treatment and surgical resection was 1.5 months (range, 0.8–10.4) for the TACE group and 3.2 months (range, 0.9–14.4) for the combination group (*p* < 0.001). The primary approach for hepatectomy in patients was open surgery, with 9 patients (10.4%) in the combination group undergoing robot‐assisted resection. The major hepatectomy rate (62.3% vs. 72.4%, *p* = 0.180) and postoperative hospital stay (9 days vs. 10 days, *p* = 0.831) showed no significant differences between the two groups. After preoperative therapy, 32 patients (46.4%) in the TACE group and 52 patients (59.8%) in the combination group achieved an objective response according to the mRECIST criteria. Postoperative pathological examination identified mVI in 19 patients (27.5%) and 17 patients (19.5%), respectively. Furthermore, pathological evaluation indicated a higher rate of complete pathological response in the combination group, although the difference was not statistically significant (8.0% vs. 2.9%, *p* = 0.171).

### 3.4 Survival Analysis for Surgical Patients

After a median post‐surgery follow‐up period of 24.0 months (range, 1.5–55.2), recurrence occurred in 101 patients (49 in the TACE group, 52 in the combination group), and 42 patients (23 in the TACE group, 19 in the combination group) died.

**FIGURE 3:**
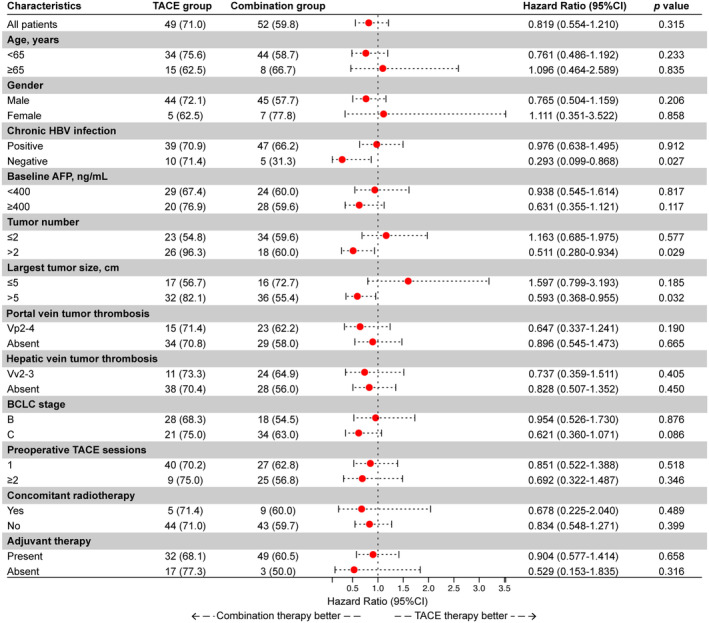
Forest plots for subgroup analysis in surgical patients. The analysis shows the hazard ratios for progression‐free survival across different subgroups. TACE, transarterial chemoembolization; HBV, hepatitis B virus; AFP, alpha‐fetoprotein; BCLC, Barcelona Clinic Liver Cancer; Vp2‐4, tumor thrombus involving the portal vein: Vp2 (second‐order branch), Vp3 (first‐order branch), Vp4 (main trunk/contralateral branch); Vv2‐3, Tumor thrombus involving the major hepatic vein (Vv2) or inferior vena cava (Vv3).

A subgroup analysis was conducted in surgical patients (shown in Figure 3). Preoperative combination therapy demonstrated a survival benefit in the following subgroups: absence of HBV infection (*p* = 0.027), largest tumor diameter > 5 cm (*p* = 0.032), and tumor number > 2 (*p* = 0.029). To more accurately identify the population benefiting from the treatment, we further analyzed up‐to‐seven criteria in the surgical cohort.

**FIGURE 4:**
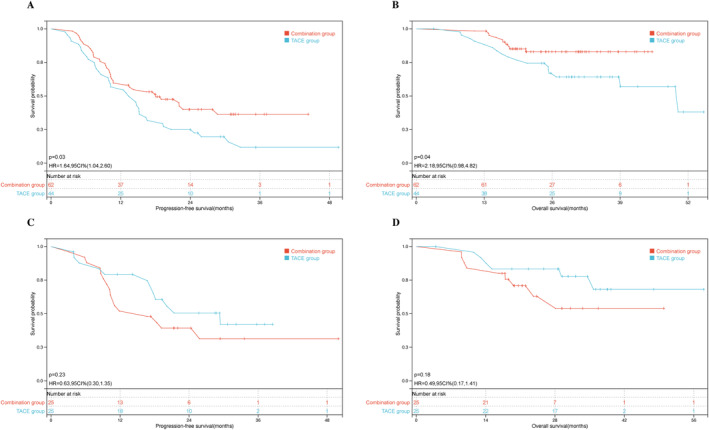
Kaplan–Meier analyses of survival outcomes of surgical patients between the TACE group and combination group within and beyond up‐to‐seven criteria. (A) Progression‐free survival in patients beyond up‐to‐seven criteria, (B) overall survival in patients beyond up‐to‐seven criteria, (C) progression‐free survival in patients within up‐to‐seven criteria, and (D) overall survival in patients within up‐to‐seven criteria. TACE, transarterial chemoembolization.

Among a total of 106 surgical patients with baseline tumor burden exceeding the up‐to‐seven criteria, PFS was statistically significantly better in the combination group compared with the TACE group (18.0 vs. 14.6 months, *p* = 0.03, HR = 0.61, 95% CI: 0.37–0.96), with corresponding 1‐year PFS rates of 59.7% and 56.8%, respectively. In addition, a significant difference in the median OS was overserved between the two groups (not reached vs. 50.1 months, *p* = 0.049, HR = 0.48, 95% CI: 0.22–1.0), and the 1‐year OS rates were 98.4% and 90.7%, respectively (shown in Figure 4A,B).

In contrast, among 50 surgical patients who met the up‐to‐seven criteria, the median PFS was 29.4 months in the TACE group and 16.7 months in the combination group, yielding a HR of 1.57 (95% CI: 0.74–3.3; *p* = 0.23), which did not reach statistical significance. Correspondingly, the 1‐year PFS rates were 52% and 79.3%, respectively. Similarly, although the median OS was not reached in either group, the TACE group showed a slightly better outcome than the combination group (not reached vs. not reached, *p* = 0.075, HR = 0.50, 95% CI: 0.18–1.40), with respective 1‐year OS rates of 95.8% and 84.0% (shown in Figure 4C,D).

The primary type of recurrence remains intrahepatic tumor recurrence. In both groups, 73.5% and 67.3% of patients, respectively, underwent a combination of locoregional therapy and systemic therapy. In the entire surgical patient cohort, Cox regression analysis for survival revealed an increased risk of worse PFS for individuals with positive chronic HBV infection (HR = 1.736, 95% CI: 1.009–3.082, *p* = 0.047), a greater baseline tumor number (HR = 1.318, 95% CI: 1.080–1.609, *p* = 0.007), a larger maximum diameter (HR = 1.092, 95% CI: 1.034–1.153, *p* = 0.002), and pathologically confirmed satellite nodules (HR = 1.692, 95% CI: 1.038–2.758, *p* = 0.035). Notably, this risk was found to be independent of the preoperative treatment modality (Table 4).

**TABLE 4:** Univariate and multivariate Cox regression analysis of PFS in the surgical cohort.

Variables	Univariate analysis (*p*)	Multivariate analysis	
		Hazard ratio (95% CI)	*p*
Treatment, TACE/combination	0.329		
Age, years, ≤ 65/> 65	0.851		
Gender, male/female	0.316		
Chronic HBV infection, positive/negative	**0.045**	**1.736 (1.009–3.082)**	**0.047**
Baseline AFP, ng/mL, < 400/≥ 400	0.171		
Tumor number^a^	**0.032**	**1.318 (1.080–1.609)**	**0.007**
Largest tumor size^a^	**0.024**	**1.092 (1.034–1.153)**	**0.002**
Portal vein tumor thrombosis, Vp2‐4/absent	0.589		
Hepatic vein tumor thrombosis, Vv2‐3/absent	0.808		
BCLC stage, B/C	0.344		
TACE sessions, 1/≥ 2	0.738		
Concomitant radiotherapy, yes/no	0.715		
Satellite nodule, present/absent	**0.004**	**1.692 (1.038–å2.758)**	**0.035**
Microvascular invasion, present/absent	0.132		
Adjuvant therapy, present/absent	0.536		

Abbreviations: AFP, alpha‐fetoprotein; BCLC, barcelona clinic liver cancer; CI, confidence interval; HBV, hepatitis B virus; PFS, progression‐free survival; TACE, transarterial chemoembolization; Vp2‐4, tumor thrombus involving the portal vein: Vp2 (second‐order branch), Vp3 (first‐order branch), Vp4 (main trunk/contralateral branch); Vv2‐3, tumor thrombus involving the major hepatic vein (Vv2) or inferior vena cava (Vv3).

Bold values indicate statistically significant *p* < 0.05.

^a^ Number or size of initially diagnosed tumors.

### 3.5 Safety

Each patient in both groups experienced at least one TRAE during the treatment period, encompassing adverse reactions related to both the TACE procedure and drug‐related effects.

The primary adverse effect following TACE was post‐embolization syndrome, characterized by abdominal pain, fever, nausea, and elevated liver function levels, with most cases being grade 1 or 2. The profile and severity of drug‐related adverse events were comparable between the surgical and non‐surgical groups. Commonly observed adverse events included fatigue, gastrointestinal reactions, skin rash, hand‐foot syndrome, thyroid dysfunction, anemia, hypoalbuminemia, and thrombocytopenia. Importantly, combination therapy did not significantly increase the incidence of grade 3/4 TRAEs compared to TACE monotherapy. In the combination group, 13 patients (14.1%) required dose reductions for anti‐angiogenic agents, and 5 patients (5.4%) experienced interruptions in systemic therapy due to intolerable toxicity, disease progression, or personal preferences.

Among surgical patients, 21 (30.4%) in the TACE group and 31 (35.6%) in the combination group experienced postoperative complications, including pleural or abdominal effusion, hepatic failure, and bile leakage. Most complications were effectively managed with symptomatic interventions. Although the overall incidence of severe complications was slightly higher in the combination group compared with the TACE group, this difference was not statistically significant. No perioperative deaths occurred in either group. Detailed TRAE profiles are summarized in Table 5.

**TABLE 5:** Treatment‐related adverse events in the surgical and non‐surgical cohorts.

AEs	Surgical patients						Non‐Surgical patients					
	Any grade, *n* (%)			Grade ≥ 3, *n* (%)			Any grade, *n* (%)			Grade ≥ 3, *n* (%)		
	TACE (*n* = 69)	Combination (*n* = 87)	*p*	TACE (*n* = 69)	Combination (*n* = 87)	*p*	TACE (*n* = 31)	Combination (*n* = 92)	*p*	TACE (*n* = 31)	Combination (*n* = 92)	*p*
Hematological AEs												
Anemia	17 (24.6)	23 (26.4)	0.798	2 (2.9)	3 (3.4)	1.000	10 (32.3)	34 (37.0)	0.637	2 (6.5)	7 (7.6)	1.000
Thrombocytopenia	15 (21.7)	19 (21.8)	0.988	2 (2.9)	2 (2.3)	1.000	7 (22.6)	24 (26.1)	0.697	3 (9.7)	9 (9.8)	1.000
Non‐hematological AEs												
Abdominal pain	30 (43.5)	39 (44.8)	0.866	4 (5.8)	6 (6.9)	1.000	18 (58.1)	65 (70.7)	0.196	4 (12.9)	13 (14.1)	1.000
ALT/AST increased	29 (42)	42 (48.3)	0.436	3 (4.3)	4 (4.6)	1.000	16 (51.6)	54 (58.7)	0.491	2 (6.5)	8 (8.7)	1.000
Fever	26 (37.7)	35 (40.2)	0.746	1 (1.4)	2 (2.3)	1.000	15 (48.4)	50 (54.3)	0.565	1 (3.2)	2 (2.2)	1.000
Fatigue	21 (30.4)	28 (32.1)	0.815	3 (4.3)	4 (4.6)	1.000	12 (38.7)	39 (42.4)	0.719	2 (6.5)	4 (4.3)	0.641
Nausea/vomiting	19 (27.5)	27 (31)	0.634	2 (2.9)	3 (3.4)	1.000	11 (35.5)	36 (39.1)	0.718	3 (9.7)	11 (12)	1.000
Hypoalbuminemia	16 (23.2)	21 (24.1)	0.890	2 (2.9)	2 (2.3)	1.000	13 (41.9)	37 (40.2)	0.866	2 (6.5)	6 (6.5)	1.000
Hyperbilirubinemia	10 (14.5)	18 (20.7)	0.316	1 (1.4)	1 (1.1)	1.000	7 (22.6)	22 (24)	0.880	1 (3.2)	4 (4.3)	1.000
Diarrhea	5 (7.2)	10 (11.5)	0.371	1 (1.4)	2 (2.3)	1.000	3 (9.7)	18 (19.6)	0.206	0	4 (4.3)	0.571
Constipation	5 (7.2)	9 (10.3)	0.501	0	0	—	2 (6.5)	10 (10.9)	0.729	0	1 (1.1)	1.000
Liver abscess	1 (1.4)	1 (1.1)	1.000	1 (1.4)	1 (1.1)	1.000	0	2 (2.2)	1.000	0	2 (2.2)	1.000
Thyroid dysfunction	0	5 (5.7)	0.067	0	1 (1.1)	1.000	0	11 (12)	0.064	0	1 (1.1)	1.000
Oral mucositis	0	5 (5.7)	0.067	0	1 (1.1)	1.000	0	11 (12)	0.064	0	2 (2.2)	1.000
Gastrointestinal bleeding	0	4 (4.6)	0.130	0	1 (1.1)	1.000	0	7 (7.6)	0.190	0	3 (3.2)	0.571
Rash	0	3 (3.4)	0.255	0	0	—	0	12 (13.0)	0.036	0	2 (2.2)	1.000
Hand‐foot syndrome	0	3 (3.4)	0.255	0	0	—	0	10 (10.9)	0.064	0	1 (1.1)	1.000
Hypertension	0	2 (2.3)	0.503	0	0	—	0	8 (8.7)	0.200	0	1 (1.1)	1.000
Hepatic encephalopathy	0	0	—	0	0	—	0	1 (1.1)	1.000	0	1 (1.1)	1.000
Perioperative complications							—	—	—	—	—	—
Pleural effusion	13 (18.8)	22 (25.3)	0.338	7 (10.1)	10 (11.5)	0.788	—	—	—	—	—	—
Ascites	9 (13)	15 (17.2)	0.470	4 (5.8)	6 (6.9)	0.781	—	—	—	—	—	—
Liver failure	3 (4.3)	4 (4.6)	1.000	2 (2.9)	2 (2.3)	1.000						
Bile leakage	2 (2.9)	3 (3.4)	1.000	1 (1.4)	1 (1.1)	1.000	—	—	—	—	—	—
Abdominal infection	2 (2.9)	2 (2.3)	1.000	1 (1.4)	2 (2.3)	1.000	—	—	—	—	—	—
Pneumonia	2 (2.9)	2 (2.3)	1.000	0	0	—	—	—	—	—	—	—
Deep vein thrombosis	1 (1.4)	3 (3.4)	0.784	0	0	—	—	—	—	—	—	—
Wound infection	1 (1.4)	1 (1.1)	1.000	0	0	—						
Hemorrhage	1 (1.4)	1 (1.1)	1.000	1 (1.4)	1 (1.1)	1.000	—	—	—	—	—	—
Renal dysfunction	0	1 (1.1)	1.000	0	1 (1.1)	1.000	—	—	—	—	—	—

Abbreviations: AEs, adverse events; ALT, alanine aminotransferase; AST, aspartate aminotransferase; TACE, transarterial chemoembolization.

## 4 Discussion

Patients with intermediate to advanced HCC continue to face significant challenges in achieving improved efficacy despite the widespread adoption of TACE. Our findings indicate that the integration of anti‐angiogenic therapy and immunotherapy with TACE leads to enhanced PFS in BCLC stage B and C non‐surgical patients, while also providing a survival benefit for surgical candidates exceeding the up‐to‐seven criteria. This combination is well‐tolerated and does not significantly elevate the risk of adverse events, underscoring its potential to optimize treatment outcomes in advanced HCC.

The observed benefits of combining TACE with systemic therapies can be attributed to their complementary mechanisms, as previously demonstrated [30, 31, 32]. Although TACE effectively reduces tumor burden, it also introduces challenges such as incomplete necrosis, hypoxia, angiogenesis, and immune suppression, which can limit its efficacy [33, 34]. The addition of anti‐angiogenic agents mitigates these issues by improving tumor revascularization and drug delivery [35]. Furthermore, ICIs potentially enhance TACE‐induced immune responses, creating a more favorable microenvironment for sustained antitumor activity [36, 37, 38, 39]. These synergistic effects likely contribute to the improved survival outcomes observed in our study. Future studies should focus on refining patient selection and identifying subgroups that derive the greatest benefit from this multimodal approach to optimize clinical outcomes.

In the present study, the combination group within the non‐surgical cohort demonstrated a prolonged median PFS of 9.4 months, which aligns with the range reported in previous studies. Published data on triple therapy for unresectable or advanced HCC indicate a median PFS ranging from 8.9 to 16.2 months [30, 40]. Notably, despite a higher proportion of patients with technically or oncologically unresectable disease—due to factors such as local tumor invasion, distant metastases, or compromised hepatic function assessed by MDTs—the non‐surgical combination group achieved superior PFS outcomes compared with those receiving TACE monotherapy. Importantly, this significant difference persisted even after PSM. Furthermore, this combination therapy regimen consistently emerged as a protective factor for PFS in both unmatched and matched cohorts, reducing the risk of disease progression by approximately 50%. These findings underscore the potential of integrating systemic therapies with TACE to deliver stronger synergistic anti‐tumor effects and sustained tumor control for patients ineligible for curative resection.

The recommendations for conversion or downstaging therapies in intermediate or advanced‐stage HCC have evolved significantly with the advent of combined strategies [40, 41]. Multiple studies have shown that combining systemic therapies with TACE can improve overall conversion rates to surgical resection compared to TACE monotherapy [16, 42, 43]. Given the complexity of tumor burden variations and the challenges of achieving effective matching between treatment groups, we conducted a subgroup analysis within the surgical cohort. The analysis revealed that patients with higher tumor burden derived significant benefits from liver resection following preoperative combination therapy. Subsequently, we performed stratified analyses based on the up‐to‐seven criteria to further refine our assessment. This stratification facilitated the categorization of patients into more homogenous subgroups, better controlling for the impact of tumor burden on survival outcomes in surgical patients.

Previous studies have demonstrated that triple combination therapy can improve disease control in patients with intermediate or advanced HCC, particularly those with tumor diameters ≤ 10 cm, more than three tumors, or distant metastases [44, 45]. Our analysis revealed that the addition of systemic therapy to TACE before surgery provides a significant tumor‐free survival advantage for patients exceeding the up‐to‐seven criteria undergoing surgical resection. In this subgroup, both PFS and OS were notably improved with the combination therapy, suggesting that this approach not only enhances disease control but also creates a more favorable environment for subsequent surgical interventions. In addition, our findings highlight that a greater baseline tumor number or larger maximum diameter was strongly associated with poorer PFS outcomes across the entire surgical cohort. This underscores the importance of baseline tumor characteristics in determining surgical outcomes and survival. Patients presenting with heavier tumor burdens at diagnosis face a heightened risk of recurrence due to factors such as aggressive tumor biology, the presence of potential intrahepatic lesions, and the limitations imposed by insufficient remnant liver volume following resection. Preoperative combination therapy addresses these challenges by effectively reducing tumor burden, thereby facilitating surgical conversion and potentially improving OS by mitigating recurrence risk and enhancing the efficacy of subsequent treatments. However, it is important to note that patients with multiple, larger, or metastatic tumors often present increased surgical complexity. These factors may partially offset the benefits of combination therapy, making it challenging to achieve consistently favorable postoperative oncological outcomes. Therefore, further exploration is warranted to determine whether combination treatments can consistently optimize the long‐term outcomes of surgical resection in these patients.

Retrospective studies have indicated that achieving a CR to TACE followed by liver resection can improve survival in intermediate‐stage HCC patients [46]. In our study, preoperative TACE in patients within the up‐to‐seven criteria demonstrated better PFS outcomes compared to combination therapy, although this difference was not statistically significant. However, several factors warrant a cautious interpretation of these findings. First, TACE has shown superior efficacy in controlling smaller intrahepatic lesions, whereas its impact on extrahepatic metastases or vascular tumor thrombus is more limited [47]. Patients with lower tumor burdens were therefore more likely to benefit from preoperative TACE, increasing the likelihood of subsequent surgical resection. For patients meeting the up‐to‐seven criteria, TACE monotherapy may already provide effective disease control, with surgery further enhancing survival outcomes [48]. As a result, combination therapy did not show additional survival benefits in this subgroup. Second, the surgical group may have included a more heterogeneous population, potentially leading to varied responses to combination therapy and masking any overall trends. Third, systemic therapies often take longer to achieve their full effect, and their benefits may be offset by the therapeutic advantages of sequential resection. Thus, the timing of surgery and the choice of postoperative treatment likely influenced the observed outcomes. Finally, the effectiveness of preoperative combination therapy may have been affected by interactions among treatment duration, resection complexity, surgical approach, and patient tolerance. These findings underscore the need for further research to determine whether systemic therapy should be used in patients within the up‐to‐seven criteria before curative resection.

In our study, no significant difference in OS was observed between non‐surgical patients initially treated with TACE or TACE combined with systemic therapies and surgical patients within the up‐to‐seven criteria who received similar initial treatments. This similarity in OS outcomes is likely attributable to the impact of subsequent treatment strategies following recurrence or progression. Guided by multidisciplinary discussions and aligned with expert consensus or established guidelines, these strategies included aggressive post‐progression interventions such as repeat hepatectomy, additional locoregional therapies, second‐line systemic treatments, and clinical trial participation. Collectively, these measures likely contributed to balancing OS outcomes between the groups. Biologically, similar OS outcomes may also reflect the intrinsic nature of advanced HCC. Rapid adaptation of HCC cells to initial treatment pressure, combined with clonal heterogeneity, can limit the long‐term impact of initial combination therapies. This highlights the inherent challenge of achieving sustained survival benefits in advanced HCC, necessitating continuous evolution and adaptation of treatment strategies. Notably, while PFS in the combination group did not show improvement among surgical patients within the up‐to‐seven criteria, the observed one‐year OS rate remained comparable to results reported in previous studies [16, 42], underscoring the importance of effective post‐progression management. These findings suggest that while combination therapy with TACE can enhance early disease control, as reflected by improved PFS, its impact on OS is more variable and heavily influenced by subsequent treatments and patient‐specific factors, such as tumor burden and surgical potential. Achieving meaningful OS benefits in advanced HCC likely requires a comprehensive, individualized approach that prioritizes both early disease control and effective post‐progression management. Future studies should focus on identifying predictive biomarkers for OS benefits and optimizing treatment sequences to improve long‐term survival outcomes across different HCC subgroups.

The optimal timing for surgical resection following preoperative therapy remains undefined. In our study, the median preoperative durations were 1.5 months in the TACE group and 3.2 months in the combination group. Early surgical resection not only reduces the risk of residual tumor progression or severe liver dysfunction but also facilitates the implementation of tailored postoperative treatment strategies based on pathological findings. Furthermore, the diverse treatment responses and survival outcomes observed in our study underscore the importance of personalized therapeutic strategies. These strategies should consider tumor characteristics, liver function, and overall patient health to maximize clinical benefits. Although the findings of this study reflect the experience of a single center and require further validation, they provide valuable insights into the integration of neoadjuvant or conventional surgical resection in patients with initially unresectable or technically resectable intermediate‐to‐advanced HCC who achieve favorable responses to preoperative therapy. These results may help guide clinical decision‐making and the optimization of treatment timing for such patients.

All TRAEs observed in this study were consistent with established safety profiles, with no unexpected overlapping toxicities. Most adverse effects were mild to moderate and manageable through dose adjustments and symptomatic treatment. No significant differences in adverse reactions were noted between the treatment groups. Notably, the incidence of gastrointestinal bleeding in the combination group was consistent with historical data from the IMbrave150 trial [49], particularly within the non‐surgical cohort. This finding underscores the importance of vigilant monitoring for patients with esophageal varices. In addition, the introduction of systemic medications did not significantly impact perioperative safety or recovery outcomes. Liver failure rates remained within acceptable levels, likely attributable to effective multidisciplinary management and advancements in surgical techniques.

Several limitations of this study should be acknowledged. First, despite applying strict inclusion criteria and conducting robust statistical analyses, this retrospective study was constrained by a limited sample size, potential subjective selection biases, and insufficient follow‐up time. Second, while efforts were made to standardize treatment protocols, the evolving complexity of TACE and surgical techniques, as well as the diversity of systemic drugs used during the study period, may have introduced variability, potentially impacting treatment consistency and outcomes. Third, although the up‐to‐seven criteria effectively stratified baseline tumor burden, they do not fully account for post‐surgical factors such as resection margin status, mVI, or adjuvant therapy, all of which could significantly influence long‐term outcomes. In addition, our study did not evaluate the relationship between radiological or pathological tumor response and long‐term outcomes. Further investigation is warranted to identify potential biomarkers predictive of antitumor response or prognosis to TACE‐based therapy and to assess the optimal application, method, and duration of postoperative adjuvant therapy. The absence of quality‐of‐life (QoL) data also represents a limitation of this study. Future research should incorporate QoL assessments, integrate additional prognostic variables to improve patient stratification and recurrence prediction, and explore biomarkers to optimize postoperative management strategies. Finally, the results from this single‐center study may limit their generalizability to broader populations or diverse healthcare settings. We also recognize that the sample size in certain subgroups may constrain the statistical power of some findings, which should be interpreted with caution. Therefore, further validation through extended follow‐up periods and large‐scale prospective studies is necessary.

## 5 Conclusion

The incorporation of anti‐angiogenic therapy and immunotherapy did not increase adverse events compared with TACE alone. Combination therapy significantly improved PFS in non‐surgical patients with BCLC B and C stage HCC and demonstrated long‐term survival benefits in surgical patients exceeding the up‐to‐seven criteria. Future research should aim to optimize combination strategies and identify predictive biomarkers to enhance personalized treatment approaches in advanced HCC.

## Author Contributions

**Chengxiang Guo:** conceptualization (equal), data curation (equal), formal analysis (equal), validation (equal), writing – original draft (equal). **Weiran Du:** conceptualization (equal), data curation (equal), formal analysis (equal), validation (equal), writing – original draft (equal). **Yiwen Chen:** data curation (equal), formal analysis (equal), investigation (equal), validation (equal). **Wenbo Xiao:** investigation (equal), supervision (equal), validation (equal). **Ke Sun:** investigation (equal), supervision (equal), validation (equal). **Yan Shen:** investigation (equal), supervision (equal), validation (equal). **Min Zhang:** investigation (equal), supervision (equal), validation (equal). **Jian Wu:** investigation (equal), supervision (equal), validation (equal). **Shunliang Gao:** investigation (equal), supervision (equal), validation (equal). **Jun Yu:** investigation (equal), supervision (equal), validation (equal). **Risheng Que:** investigation (equal), supervision (equal), validation (equal). **Xing Xue:** investigation (equal), supervision (equal), validation (equal). **Xueli Bai:** conceptualization (equal), investigation (equal), methodology (equal), supervision (equal), writing – review and editing (equal). **Tingbo Liang:** conceptualization (equal), investigation (equal), methodology (equal), supervision (equal), writing – review and editing (equal).

## Ethics Statement

As this study is a retrospective review, it did not involve any prospective data collection from human subjects. Therefore, formal ethics approval was not required for this research. The study design and data analysis adhered to ethical standards and guidelines. This exemption from ethics approval was determined by the research ethics committee of the First Affiliated Hospital of Zhejiang University. Informed consent was waived for this retrospective study as it involves the analysis of existing data and does not compromise the welfare and rights of the patients. The utilization of non‐identified information ensures the protection of patient confidentiality. This waiver was in accordance with the decision made by the research ethics committee of the First Affiliated Hospital of Zhejiang University and conforms to ethical standards for retrospective research.

## Conflicts of Interest

The authors declare no conflicts of interest.

## Supporting information


**Figure S1.** Kaplan–Meier analyses of survival outcomes for the entire cohort (surgical and non‐surgical patients) comparing TACE and combination groups before and after propensity score matching (PSM). (A) Progression‐free survival before PSM, (B) overall survival before PSM, (C) progression‐free survival after PSM, and (D) overall survival after PSM. TACE, transarterial chemoembolization.

**Table S1.** Detailed treatment information for non‐surgical patients.

**Table S2.** Detailed treatment information and oncological outcomes for surgical patients.

**Table S3.** Clinical characteristics of the patient cohort.

## Data Availability

All data generated or analyzed during this study are included in this article and its Supporting Information files. Further enquiries can be directed to the corresponding author.
