# Supplementary figures and images for: Transarterial Chemoembolization With or Without Systemic Therapy for Unresectable Hepatocellular Carcinoma: A Retrospective Comparative Study
Source: Cancer Med. 2025 Feb 5;14(3):e70633. doi: 10.1002/cam4.70633 (PMC11795419; doi:10.1002/cam4.70633)

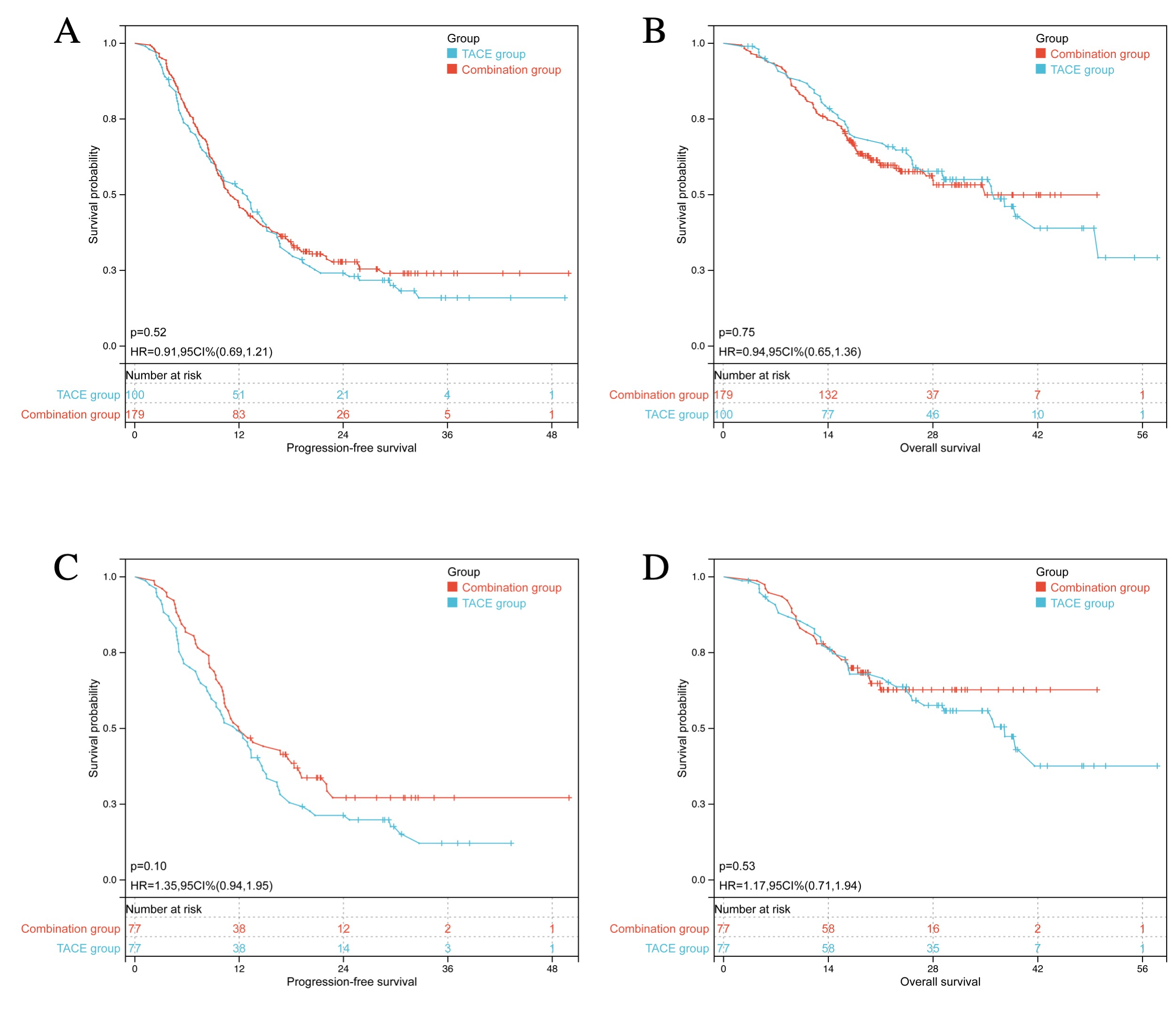

Supplement: Supplementary file 1 — Figure S1. Kaplan–Meier analyses of survival outcomes for the entire cohort (surgical and non‐surgical patients) comparing TACE and combination groups before and after propensity score matching (PSM). (A) Progression‐free survival before PSM, (B) overall survival before PSM, (C) progression‐free survival after PSM, and (D) overall survival after PSM. TACE, transarterial chemoembolization. [file CAM4-14-e70633-s001.tiff]
