# Supplementary material for: Transarterial Chemoembolization With or Without Systemic Therapy for Unresectable Hepatocellular Carcinoma: A Retrospective Comparative Study
Source: Cancer Med. 2025 Feb 5;14(3):e70633. doi: 10.1002/cam4.70633 (PMC11795419; doi:10.1002/cam4.70633)
Supplement: Supplementary file 2 — Table S1. Detailed treatment information for non‐surgical patients. [file CAM4-14-e70633-s002.docx]

**Supplemental Table 1** Detailed treatment information for non-surgical patients

|  | General cohort | | | PSM cohort | | |
| --- | --- | --- | --- | --- | --- | --- |
| Characteristics | TACE  (n=31) | Combination (n=92) | *p* value | TACE  (n=26) | Combination (n=26) | *p* value |
| TACE sessions, median (IQR) | 2 (1,2) | 2 (1,3) | 0.684 | 2 (1,2.8) | 1.5 (1,2) | 0.225 |
| Total TACE |  |  | 0.647 |  |  | 0.959 |
| cTACE | 44 (69.8) | 142 (72.8) |  | 38 (69.1) | 32 (69.6) |  |
| DEB-TACE | 19 (30.2) | 53 (27.2) |  | 17 (30.9) | 14 (30.4) |  |
| Antiangiogenic drugs |  |  | - |  |  | - |
| Lenvatinib | - | 72 (78.3) |  | - | 18 (69.2) |  |
| Sorafenib | - | 12 (13.0) |  | - | 5 (19.2) |  |
| Donafenib | - | 5 (5.4) |  | - | 2 (7.7) |  |
| Bevacizumab | - | 3 (3.3) |  | - | 1 (3.8) |  |
| ICIs |  |  | - |  |  | - |
| Tislelizumab | - | 27 (29.3) |  | - | 4 (15.4) |  |
| Sintilimab | - | 23 (25.0) |  | - | 9 (34.6) |  |
| Envolizumab | - | 19 (20.7) |  | - | 6 (23.1) |  |
| Camrelizumab | - | 9 (9.8) |  | - | 2 (7.7) |  |
| Toripalimab | - | 8 (8.7) |  | - | 1 (3.8) |  |
| Pembrolizumab | - | 3 (3.3) |  | - | 2 (7.7) |  |
| Atezolizumab | - | 2 (2.2) |  | - | 1 (3.8) |  |
| Nivolumab | - | 1 (1.1) |  | - | 1 (3.8) |  |
| Duration of treatments, median (IQR), month | 6.1 (4.1–11.4) | 8.4 (4.9–14.7) | 0.050 | 6.8 (4.6–11.9) | 7.7 (4.3–11.6) | 0.701 |
| Type of progression |  |  |  |  |  |  |
| Intrahepatic disease progression | 24 (80.0) | 68 (88.3) | 0.140 | 20 (80.0) | 19 (95.0) | 0.173 |
| Extrahepatic metastasis | 5 (16.7) | 4 (5.2) |  | 4 (16.0) | 0 |  |
| Both | 1 (3.3) | 5 (6.5) |  | 1 (4.0) | 1 (5.0) |  |
| Treatment after progression |  |  | 0.316 |  |  | 0.421 |
| Systemic therapy | 5 (16.7) | 16 (20.8) |  | 3 (12.0) | 1 (5.0) |  |
| TACE+systemic therapy | 17 (56.7) | 26 (33.8) |  | 17 (68.0) | 11 (55.0) |  |
| SBRT/RFA+systemic therapy | 1 (3.3) | 4 (5.2) |  | 1 (4.0) | 1 (5.0) |  |
| HAIC+systemic therapy | 0 | 4 (5.2) |  | 0 | 0 |  |
| Clinical trial | 0 | 7 (9.1) |  | 0 | 2 (10.0) |  |
| Best supportive therapy | 7 (23.3) | 20 (25.9) |  | 4 (16.0) | 5 (25.0) |  |

**Abbreviations:** PSM, Propensity Score Matching; TACE, Transarterial Chemoembolization; cTACE, Conventional Transarterial Chemoembolization; DEB-TACE, Drug-Eluting Bead Transarterial Chemoembolization; ICIs, Immune Checkpoint Inhibitors; SBRT, Stereotactic Body Radiotherapy; RFA, Radiofrequency Ablation; HAIC, Hepatic Arterial Infusion Chemotherapy; IQR, Interquartile Range.

.
