# Supplementary material for: Transarterial Chemoembolization With or Without Systemic Therapy for Unresectable Hepatocellular Carcinoma: A Retrospective Comparative Study
Source: Cancer Med. 2025 Feb 5;14(3):e70633. doi: 10.1002/cam4.70633 (PMC11795419; doi:10.1002/cam4.70633)
Supplement: Supplementary file 3 — Table S2. Detailed treatment information and oncological outcomes for surgical patients. [file CAM4-14-e70633-s003.docx]

**Supplemental Table 2** Detailed treatment information and oncological outcomes for surgical patients

| Characteristics | TACE  (n=69) | Combination  (n=87) | *p* value |
| --- | --- | --- | --- |
| TACE sessions, median (IQR) | 1 (1,1) | 2 (1,2) | < 0.001 |
| Total preoperative TACE |  |  |  |
| cTACE | 53 (63.9) | 107 (77.0) |  |
| DEB-TACE | 30 (36.1) | 32 (23.0) |  |
| Antiangiogenic drugs |  |  | - |
| Lenvatinib | - | 69 (79.3) |  |
| Sorafenib | - | 8 (9.2) |  |
| Bevacizumab | - | 6 (6.9) |  |
| Apatinib | - | 4 (4.6) |  |
| ICIs |  |  | - |
| Sintilimab | - | 31 (35.6) |  |
| Tislelizumab | - | 19 (21.8) |  |
| Envolizumab | - | 17 (19.5) |  |
| Camrelizumab | - | 15 (17.2) |  |
| Toripalimab | - | 4 (4.6) |  |
| Atezolizumab | - | 1 (1.1) |  |
| Duration of treatments, median (IQR), month | 1.5 (1.4–1.8) | 3.2 (1.9–4.9) | < 0.001 |
| Best response by mRECIST |  |  |  |
| Complete response | 4 (5.8) | 7 (8.1) |  |
| Partial response | 28 (40.6) | 45 (51.7) |  |
| Stable disease | 37 (53.6) | 35 (40.2) |  |
| Surgical type |  |  |  |
| Open | 52 (75.4) | 51 (58.6) |  |
| Laparotomy | 17 (24.6) | 27 (31.0) |  |
| Robot-assisted | 0 | 9 (10.4) |  |
| Major hepatectomy | 43 (62.3) | 63 (72.4) | 0.180 |
| Blood loss, median (IQR), ml | 200 (200,325) | 200 (100,400) | 0.284 |
| Blood transfusion | 17 (24.6) | 23 (26.4) |  |
| Postoperative hospital day, Median, (IQR), days | 9 (7,13) | 10 (7,13) | 0.831 |
| 30-day Readmission | 1 (1.4) | 1 (1.1) |  |
| Satellite nodule | 13 (18.8) | 14 (16.1) |  |
| Microscopic vascular invasion | 19 (27.5) | 17 (19.5) |  |
| R0 resection | 69 (100) | 87 (100) | 1.000 |
| Pathologic complete response | 2 (2.9) | 7 (8.0) | 0.171 |
| Type of recurrence |  |  | 0.425 |
| Intrahepatic recurrence | 44 (89.8) | 44 (84.6) |  |
| Extrahepatic metastasis | 4 (8.2) | 4 (7.7) |  |
| Both | 1 (2.0) | 4 (7.7) |  |
| Treatment after recurrence |  |  | 0.650 |
| Systemic therapy | 5 (10.2) | 8 (15.4) |  |
| TACE+systemic therapy | 26 (53.1) | 23 (44.2) |  |
| SBRT/RFA+systemic therapy | 10 (20.4) | 12 (23.1) |  |
| Re-resection | 5 (10.2) | 3 (5.8) |  |
| Best supportive therapy | 3 (6.1) | 6 (11.5) |  |

**Abbreviations:** TACE, Transarterial Chemoembolization; cTACE, Conventional Transarterial Chemoembolization; DEB-TACE, Drug-Eluting Bead Transarterial Chemoembolization; ICIs, Immune Checkpoint Inhibitors; mRECIST, Modified Response Evaluation Criteria in Solid Tumors; SBRT, Stereotactic Body Radiotherapy; RFA, Radiofrequency Ablation; IQR, Interquartile Range.
