# Supplementary material for: Transarterial Chemoembolization With or Without Systemic Therapy for Unresectable Hepatocellular Carcinoma: A Retrospective Comparative Study
Source: Cancer Med. 2025 Feb 5;14(3):e70633. doi: 10.1002/cam4.70633 (PMC11795419; doi:10.1002/cam4.70633)
Supplement: Supplementary file 4 — Table S3. Clinical characteristics of the patient cohort. [file CAM4-14-e70633-s004.docx]

**Supplemental Table 3** Clinical characteristics of the patient cohort.

|  | General | | | PSM | | |
| --- | --- | --- | --- | --- | --- | --- |
| Characteristics | TACE  (n=100) | Combination (n=179) | *p* Value | TACE  (n=77 | Combination (n=77) | *p* Value |
| Age, years, median (IQR) | 62 (53–67) | 55 (47–60) | <0.001 | 60 (50–67) | 59 (51–65) | 0.417 |
| Gender n. (%) |  |  | 0.365 |  |  | 0.291 |
| Male | 87 (87.0) | 162 (90.5) |  | 67 (87.0) | 71 (92.2) |  |
| Female | 13 (13.0) | 17 (9.5) |  | 10 (13.0) | 6 (7.8) |  |
| Chronic HBV infection |  |  | 0.816 |  |  | 1.000 |
| Positive | 81 (81.0) | 147 (82.1) |  | 61 (79.2) | 61 (79.2) |  |
| Negative | 19 (19.0) | 32 (17.9) |  | 16 (20.8) | 16 (20.8) |  |
| Baseline AFP, ng/mL |  |  | 0.013 |  |  | 0.621 |
| ≥400 | 37 (37.0) | 94 (52.5) |  | 32 (41.6) | 29 (37.7) |  |
| <400 | 63 (63.0) | 85 (47.5) |  | 45 (58.4) | 48 (62.3) |  |
| Child Pugh |  |  | 0.454 |  |  | 0.731 |
| A | 95 (95.0) | 165 (92.7) |  | 72 (93.5) | 73 (94.8) |  |
| B | 5 (5.0) | 13 (7.3) |  | 5 (6.5) | 4 (5.2) |  |
| Tumor number* |  |  | 0.717 |  |  | 0.871 |
| ≤2 | 57 (57.0) | 98 (54.7) |  | 43 (55.8) | 42 (54.5) |  |
| >2 | 43 (43.0) | 81 (45.3) |  | 34 (44.2) | 35 (45.5) |  |
| Largest tumor size*, cm, median (IQR) | 6.4 (4.1–9.3) | 8.3 (5.0–11.8) | <0.001 | 6.5 (4.1–9.7) | 6.5 (4.1–10.1) | 0.712 |
| Portal vein tumor thrombosis |  |  | 0.007 |  |  | 0.511 |
| Vp2-4 | 38 (38.0) | 98 (54.7) |  | 29 (37.7) | 33 (42.9) |  |
| Absent | 62 (62.0) | 81 (45.3) |  | 48 (62.3) | 44 (57.1) |  |
| Hepatic vein tumor thrombosis |  |  | <0.001 |  |  | 0.564 |
| Vv2-3 | 20 (20.0) | 74 (41.3) |  | 19 (24.7) | 16 (20.8) |  |
| Absent | 80 (80.0) | 105 (58.7) |  | 58 (75.3) | 61 (79.2) |  |
| Extrahepatic metastasis |  |  | 0.016 |  |  | 0.440 |
| Present | 7 (7.0) | 31 (17.3) |  | 7 (9.1) | 10 (13.0) |  |
| Absent | 93 (93.0) | 148 (82.7) |  | 70 (90.9) | 67 (87.0) |  |
| BCLC stage |  |  | <0.001 |  |  | 0.628 |
| B | 51 (51.0) | 55 (30.7) |  | 38 (49.4) | 35 (45.5) |  |
| C | 49 (49.0) | 124 (69.3) |  | 39 (50.6) | 42 (54.5) |  |
| TACE sessions |  |  | <0.001 |  |  | 0.746 |
| 1 | 67 (67.0) | 43 (49.4) |  | 44 (57.1) | 42 (54.5) |  |
| ≥2 | 33 (33.0) | 44 (50.6) |  | 33 (42.9) | 35 (45.5) |  |
| Concomitant radiotherapy |  |  | 0.109 |  |  | 0.197 |
| Yes | 13 (13.0) | 37 (20.7) |  | 10 (13.0) | 16 (20.8) |  |
| No | 87 (87.0) | 142 (79.3) |  | 67 (87.0) | 61 (79.2) |  |
| Surgical resection |  |  | <0.001 |  |  | 0.503 |
| Yes | 69 (69.0) | 87 (48.6) |  | 47 (61.0) | 51 (66.2) |  |
| No | 31 (31.0) | 92 (51.4) |  | 30 (39.0) | 26 (33.8) |  |

*Number or size of preoperatively diagnosed tumors.

**Abbreviations:** PSM, Propensity Score Matching; TACE, Transarterial Chemoembolization; HBV, Hepatitis B Virus; AFP, Alpha-Fetoprotein; BCLC, Barcelona Clinic Liver Cancer; Vp2-4, Tumor thrombus involving the portal vein: Vp2 (second-order branch), Vp3 (first-order branch), Vp4 (main trunk/contralateral branch); Vv2-3, Tumor thrombus involving the major hepatic vein (Vv2) or inferior vena cava (Vv3); IQR, Interquartile Range.
